# SOX11 expression correlates to promoter methylation and regulates tumor growth in hematopoietic malignancies

**DOI:** 10.1186/1476-4598-9-187

**Published:** 2010-07-12

**Authors:** Elin Gustavsson, Sandra Sernbo, Elin Andersson, Donal J Brennan, Michael Dictor, Mats Jerkeman, Carl AK Borrebaeck, Sara Ek

**Affiliations:** 1 Department of Immunotechnology, Lund University, Lund Sweden; 2 CREATE Health, Lund University, BMC D13, 221 84, Lund Sweden; 3 UCD School of Biomolecular and Biomedical Science, UCD Conway Institute, University College Dublin, Ireland; 4 Department of Pathology, Lund University Hospital, Lund Sweden; 5 Department of Oncology, Lund University Hospital, Lund Sweden

## Abstract

**Background:**

The transcription factor SOX11 plays an important role in embryonic development of the central nervous system (CNS) and is expressed in the adult immature neuron but is normally not expressed in any other adult tissue. It has recently been reported to be implicated in various malignant neoplasms, including several lymphoproliferative diseases, by its specific expression and in some cases correlation to prognosis. SOX11 has been shown to prevent gliomagenesis *in vivo *but the causes and consequences of aberrant expression of *SOX11 *outside the CNS remain unexplained.

**Results:**

We now show the first function of *SOX11 *in lymphoproliferative diseases, by demonstrating *in vitro *its direct involvement in growth regulation, as assessed by siRNA-mediated silencing and ectopic overexpression in hematopoietic malignancies. Gene Chip analysis identified cell cycle regulatory pathways, including Rb-E2F, to be associated with SOX11-induced growth reduction. Furthermore, promoter analysis revealed that *SOX11 *is silenced through DNA methylation in B cell lymphomas, suggesting that its regulation is epigenetically controlled.

**Conclusions:**

The data show that SOX11 is not a bystander but an active and central regulator of cellular growth, as both siRNA-mediated knock-down and ectopic overexpression of *SOX11 *resulted in altered proliferation. Thus, these data demonstrate a tumor suppressor function for *SOX11 *in hematopoietic malignancies and revealed a potential epigenetic regulation of this developmentally involved gene.

## Background

The neural transcription factor SOX11 is a novel diagnostic antigen for mantle cell lymphoma (MCL) [1]. However, the prognostic relevance of nuclear expression of SOX11 in MCL remains unclear since it has been associated with both improved and reduced survival [2,3]. Our recent investigations demonstrated that nuclear staining of SOX11 is also seen in Burkitt Lymphoma (BL) and precursor B and T cell lymphoblastic neoplasia [4], indicating a more widespread role in lymphoproliferative diseases than initially anticipated as also confirmed by others [5]. Furthermore, analysis of solid tumors revealed a strong nuclear expression of SOX11 in epithelial ovarian cancer (EOC), which correlated with a prolonged recurrence-free survival [6], suggesting a functional role for SOX11 in regulation of tumor growth. Abundant *SOX11 *expression has been described in both the fetal central nervous system (CNS) and CNS-derived malignancies, such as medulloblastoma [7] and malignant glioma [8]. Furthermore, overexpression of SOX11 has been shown to prevent tumorigenesis in human glioma initiating cells [9]. However, our previous study on EOC demonstrated that SOX11 also might be involved in growth regulation of malignancies not related to the CNS [6].

SOX11 belongs to a group of 20 transcription factors within the high-mobility group (HMG) box protein super family, which are characterized by high sequence homology within their DNA-binding HMG domain [10]. It has been shown that this HMG domain serves two functions, i.e. DNA binding as well as partner selection, which may permit selective recruitment of SOX proteins to specific promoters and transcription factors [11-13]. To date, the main function of SOX11 in non-malignant tissues has been its involvement in neural development [14,15] and organogenesis [13] during fetal development. Recent data also suggest an important role for SOX11 as a transcriptional regulator in adult immature neurons [16].

The correlation between SOX11 and differences in survival in MCL [2,3] and EOC [6] lead us to further investigate the mechanisms underlying its expression. In the present study, we used functional and epigenetic analyses of B cell malignancies to demonstrate a regulatory mechanism of *SOX11 *expression on tumor cell growth. In conclusion, we provide the first evidence of a growth regulatory role for SOX11 outside the CNS. Furthermore, this protein not only has a tumor suppressor function but is also epigenetically silenced through DNA methylation in a subset of B cell malignancies.

## Results

### Analysis of DNA methylation of the *SOX11 *promoter region

**Figure 1:**
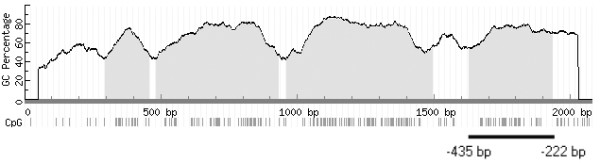
**CpG islands in the *SOX11 *promoter region**. Analysis of 2000 bp upstream of *SOX11 *transcription start revealed four CpG islands with a GC content above 50 percent http://www.urogene.org/methprimer/index1.html [45]. CpG dinucleotides are represented as vertical bars. Primers that amplified -435 to -222 were used in bisulfite sequencing to compare the methylation status of the *SOX11 *promoter region with *SOX11 *expression.

Epigenetic regulation of *SOX11 *expression was investigated by studying promoter methylation. Analysis of the 2000 bp region upstream of the transcription start site of *SOX11 *identified four CpG islands (Figure 1). DNA hypermethylation of such islands is a common event in tumor progression and leads to silencing of the corresponding gene [17]. The methylation status of the *SOX11 *promoter was assessed in nineteen cell lines, originating from different B cell malignancies, including eight MCL, three DLBCL, four FL, three BL and one acute monocytic leukemia (MONO-L) (Table 1). Initially, seven cell lines were assessed for presence of methylated CpGs in all four identified CpG islands (Figure 1). However only the CpG island closest to transcription start was determinative for *SOX11 *expression in all seven cell lines (data not shown), thus in the remaining analyses bisulfite sequencing was performed on the CpG island adjacent to the *SOX11 *transcription start site, covering 28 unique CpG sites (Figure 1).

**Table 1:** Cell lines included in the study and type of method for DNA methylation analysis.

Cell line*	Lymphoma**	Supplier	DNA methylation analysis
GRANTA-519	MCL	DSMZ	D,T
SP53	MCL	*****	D,T
Z138	MCL	****	D,T
HBL-2	MCL		D
JEKO-1	MCL	DSMZ	D
JVM-2	MCL	DSMZ	D
REC-1	MCL	DSMZ	D
UPN-2	MCL		D,T
WSU-NHL	FL	DSMZ	D,T
SC-1	FL	DSMZ	D
RL	FL	DSMZ	D
DOHH-2	FL	DSMZ	D
SU-DHL-8	DLBCL	***	D,T
ULA	DLBCL	***	D
KARPAS	DLBCL	***	D
RAMOS	BL	DSMZ	D
RAJI	BL	DSMZ	D
DAUDI	BL	DSMZ	D
THP-1	MONO-L	DSMZ	D

*The *SOX11 *gene was sequenced in all cell lines

** see text for abbreviations

*** Kindly provided by Dr Kristina Drott, Lund University

**** Kindly provided by Dr Martin Dyer at Leicester University

***** Kindly provided by Dr Mats Ehinger, Lund University

D direct sequencing

T TOPO-TA cloning of individual alleles

DSMZ, Deutsche Sammlung von Mikroorganismen und Zellkulturen GmbH

**Figure 2:**
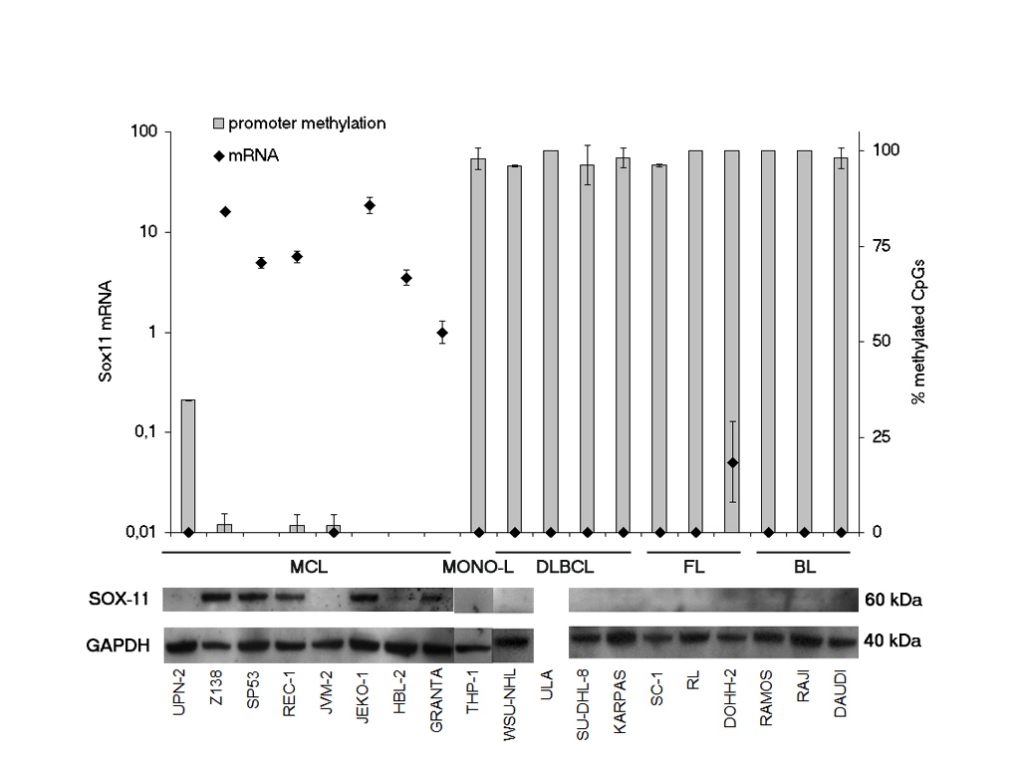
**Methylation status of *SOX11 *promoter region correlated to *SOX11 *expression**. Methylation status of SOX11 promoter (described as percentage of methylated CpGs of 28 possible CpG methylation sites) was analyzed by direct bisulfite sequencing (right Y-axis) and correlated to SOX11 expression on mRNA (left Y-axis) and protein level in nineteen lymphoid or monocytic cell lines (Table 1). Generally, all samples with a ΔC_T _(SOX11+RT, SOX11-RT) < |2| was considered negative and the RQ was set to 0.01 for those samples. RQ values are related to the SOX11 expression in GRANTA-519 and the error bars show the 95% confidence interval

A striking difference in *SOX11 *promoter methylation was detected between MCL and non-MCL lymphoma cell lines (Figure 2). The results were confirmed on individual alleles with TOPO-TA cloning for seven of the cell lines (Table 1). Analysis of non-MCL cell lines revealed high levels of *SOX11 *promoter methylation in all cases (11/11), corresponding to a lack of both *SOX11 *mRNA and protein expression (Figure 2). In contrast, *SOX11 *promoter methylation was absent in the majority (7/8) of MCL-derived cell lines, with *SOX11 *mRNA and protein expression being evident in 6 of the cell lines (GRANTA-519, HBL-2, JEKO-1, REC-1, SP53 and Z138) (Figure 2). UPN-2 was partially methylated, and lacks *SOX11 *expression. JVM-2 was the only MCL cell line lacking *SOX11 *mRNA and protein, although the promoter was not methylated in any of the 28 CpG's investigated.

Untreated clinical specimens were collected to assess the methylation status in primary material from MCL (n = 4), FL (n = 5) and a single case of DLBCL (see Additional File 1: Table S1). To enable specific methylation analysis, the B cells were purified, using positive selection on CD19-coated beads, and purity was assessed, using flow cytometry analysis (see Additional File 1: Table S1). Thus, the analyzed samples contained predominantly B cells, however, the frequency of tumor B cells will vary between entities.

**Figure 3:**
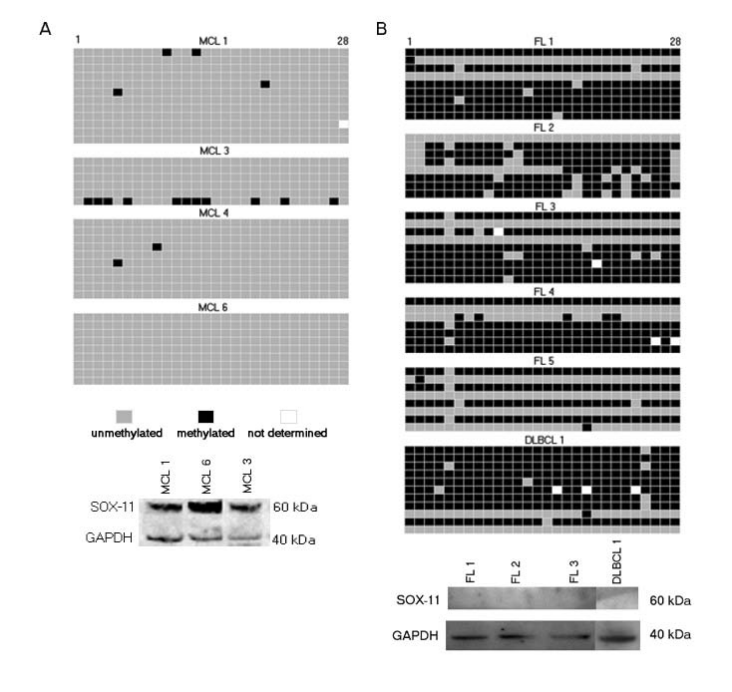
***SOX11 *DNA methylation and protein expression in primary lymphoma samples**. Methylation patterns of *SOX11 *promoter in clinical specimens was determined by bisulfite sequencing of individual alleles and correlated to SOX11 protein expression. Every row represents a unique allele and the columns represent a potentially methylated CpG site. a) In MCL samples, the promoter stays unmethylated and SOX11 is detectable. b) The lack of SOX11 protein in FL and DLBCL is accompanied by 50-100% methylated alleles.

In agreement with the *in vitro *data a difference in DNA methylation status of the *SOX11 *promoter was evident between expressing and non-expressing primary B cell lymphomas. The *SOX11 *promoter was not methylated in primary MCL (Figure 3A), which was consistent with protein analysis (Figure 3A lower panel). However, extensive (>70%) DNA methylation was seen in the DLBCL and FL samples, apart from FL5 where less (50%) of the alleles were methylated (Figure 3B, upper panel), possibly due to a sub-population of non-malignant cells. As expected, none of the primary FL or DLBCL samples expressed the SOX11 protein (Figure 3B, lower panel). This is in agreement with both previous gene expression data [1] and current RT-PCR data on DLBCL cell lines (Figure 2) and thus confirms that the cytoplasmic staining seen in IHC for DLBCL and other non-MCLs [1,3-5,18] should not be considered to be specific for SOX11.

Consequently, the lack of *SOX11 *promoter methylation in MCL compared to the methylated FL and DLBCL samples, points towards methylation-mediated *SOX11 *silencing in B cell malignancies.

### *SOX11 *knockdown in MCL cell lines results in increased cellular proliferation

**Figure 4:**
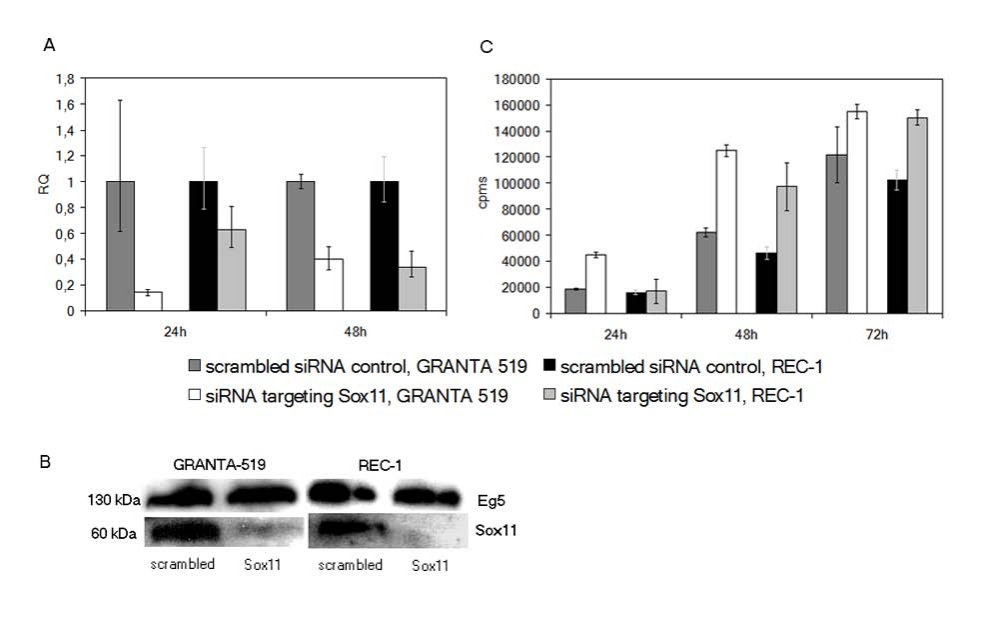
**siRNA knock-down of *SOX11 *increase proliferation**. Effect of the siRNA induced knock-down of the *SOX11 *gene in GRANTA-519 and REC-1 on, a) mRNA level at 24 and 48 h; b) protein level at 48 h and 72 h, respectively, and c) proliferation at 24, 48 and 72 h. A control siRNA targeting the Eg5 gene was used as a positive control (only shown in b). All values in a) are relative quantity values (RQ) compared to the scrambled siRNA control. The data is representative of three independent assays. In a) the error bars show the 95% confidence interval, while in c) ±1 SD is shown.

The functional effect of SOX11 was then investigated in well-characterized *in vitro *models of MCL (GRANTA-519 and REC-1). siRNA mediated gene silencing (see Additional File 1: Table S3) resulted in a significant decrease of both *SOX11 *mRNA (Figure 4A) and protein (Figure 4B) and lead to an almost 100% increase in proliferation at 48 hrs (Figure 4C). The follicular lymphoma cell line did not express any SOX11 and no change in proliferation was consequently detected (data not shown). The effect on mRNA expression reached a maximum decrease at 24 hrs for GRANTA-519 and at 48 h for REC-1 (Figure 4A), while the subsequent decrease in protein level was most pronounced at 72 h for GRANTA-519 and at 48 h for REC-1 (Figure 4B). The functional effect on cell proliferation seen at 48 h for both MCL cell lines (Figure 4C), demonstrates a growth regulatory role for SOX11.

### SOX11 overexpression in cell lines inhibits proliferation

**Figure 5:**
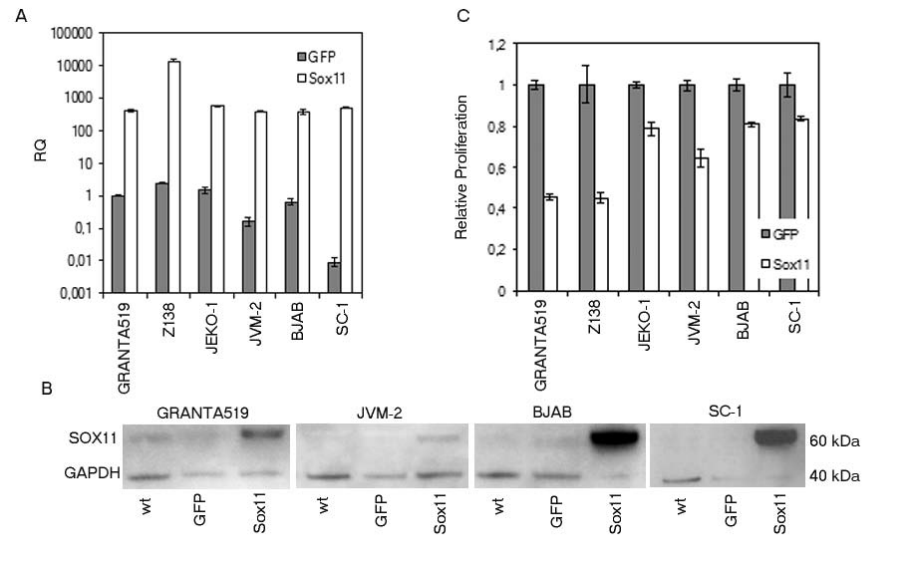
**Overexpression of *SOX11 *decrease proliferation**. a) mRNA expression of *SOX11 *at 24 h after overexpression of the *SOX11 *gene in six B cell lymphoma cell lines. b) Western blot analysis at 24 h confirms *SOX11 *overexpression in *SOX11 *transfected samples (right), compared to wt (left) and control vector (middle), loading control (GAPDH) is seen below c) Proliferation assay at 48 h after transfection showed decreased cell growth in all cell lines but BJAB where the decrease could be seen already after 24 h. In a) all values are relative quantity (RQ) compared to the GFP value for GRANTA 519. In c) all cell lines are compared to their respective GFP value. The data is representative of three independent assays. In a) the error bars show the 95% confidence interval, while in c) ±1SD is shown.

To further investigate the direct effect of SOX11 regulation, transient over-expression of SOX11 in a panel of cell lines was performed (see Table 1). *SOX11 *cDNA (see Additional File 1: Table S2) and a control vector containing GFP were introduced via nucleofection. Cell viability was 80-95% in all cell lines 24-48 h after nucleofection of both control and *SOX11 *plasmid (data not shown). Different levels of mRNA overexpression were evident at 24 h (Figure 5A) in both SOX11 negative (SC-1, JVM-2) and positive (BJAB, JEKO-1, GRANTA-519 and Z138) cell lines, resulting in different expression of SOX11 protein (Figure 5B). However, no direct correlation between mRNA and protein levels could be seen, in fact BJAB and SC-1 showed strongest increase in protein level (Figure 5B), although the amount of *SOX11 *mRNA was similar in all cell lines but Z138. The proliferation at 24 and 48 h clearly indicated that all cell lines grew significantly slower due to the SOX11 overexpression, with the most pronounced effect at 48 h for all cell lines but BJAB, in which decreased proliferation was seen already at 24 h (Figure 5C). The strongest reduction in proliferation was seen for GRANTA-519, Z138 and JVM-2, the latter being SOX11-negative in wt cells (Figure 5C). Thus, SOX11 directly regulates growth in all cell lines analyzed, independent of their original SOX11 status.

### Global gene expression analysis identified the Rb-E2F growth regulatory pathway to be involved in SOX11-related growth reduction

Both siRNA-mediated knock-down and forced overexpression of SOX11 result in change in proliferation of target cells (Figure 4c and 5c). To understand the basic mechanisms involved in this macroscopic event, global gene expression analysis of SOX11-overexpressing and control cells was performed using the MCL cell lines GRANTA-519 and JEKO-1.

**Figure 6:**
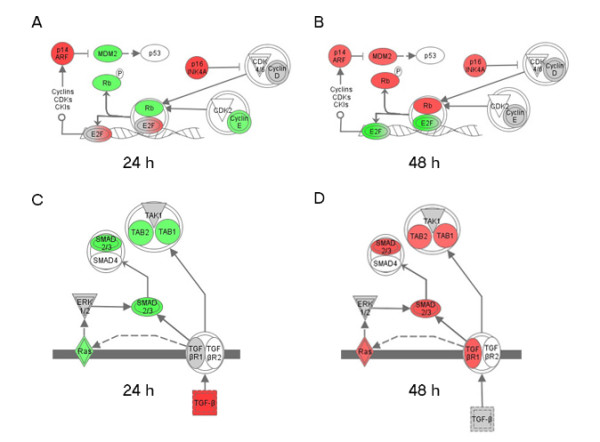
**Gene Chip analysis reveal SOX11-induced regulation of Rb-E2F and TGF-β signaling pathways**. Ingenuity Pathway Analysis identified the canonical pathway "Molecular Mechanisms in Cancer" as highly associated with the 3647 deregulated genes. Within this pathway RB-E2F (A and B) and TGF-β (C and D) signaling is regulated in a time-dependent manner as shown after 24 h (A and C) and 48 h (B and D) of ectopic SOX11-overexpression. The differentially regulated genes are marked in red or green when the mean fold change for GRANTA-519 and JEKO-1 was ≥1.2 or ≤1.2. The remaining differentially regulated genes, including genes with different kinetics in the two cell lines, are marked in grey.

4861 transcripts (see Additional File 2) were found to be differentially expressed in GRANTA-519 and JEKO-1 at either 24 or 48 h of ectopic SOX11-overexpression. These genes and corresponding mean fold change values for 24 and 48 h of SOX11-overexpression were imported into Ingenuity Pathway Analysis (IPA, Ingenuity Systems, Mountain view, CA). IPA analysis (Ingenuity Systems) recognized 3647 individual genes among the 4861 GeneChip transcripts and identified "cell cycle" as the main molecular function associated with SOX11-induced genes. The identified cell cycle-associated transcripts (n = 355) are marked with an asterisk in Additional File 2. The strong correlation between the differentially regulated genes and cell cycle function is consistent with the observed change in proliferation (Figure 5c) emphasizing the biological relevance of the differentially regulated genes in relation to MCL and SOX11. Furthermore, IPA analysis was used to identify the main canonical pathway associated with the differentially regulated genes. The identified pathway, "Molecular Mechnisms in Cancer", is shown in full in Supplementary Figure S1 for 24 h (S1a) and 48 h (S1b) of ectopic SOX11-overexpression. Sections of the canonical pathway are shown in Figure 6a-d. Among others, this includes the Rb-E2F signaling pathway, known to be of major importance in MCL [19].

Of interest, up-regulation of the CDKN2A locus, including p16^INK4A ^and p14^ARF^, and TGF-β at 24 h may promote the change in gene expression seen comparing samples after 24 and 48 h of SOX11-overexpression. At 48 h, up-regulation of anti-proliferative genes, including BRCA1 and Smad2/3, and the down-regulation of E2F1 (Figure 6b) is consistent with the decrease in proliferation seen for GRANTA-519 and JEKO-1 at this time point (Figure 5c). The differentially regulated genes are marked in red or green corresponding to a mean fold change of ≥1.2 or ≤1.2, respectively as shown for 24 h (Figure 6a) and 48 h (Figure 6b) of SOX11-overexpression. The remaining differentially regulated genes are marked in grey. The latter represent genes that have different kinetics or regulation in GRANTA-519 and JEKO-1, being up or down-regulated at either 24 or 48 h of SOX11-overexpression.

In all, Gene Chip analyses demonstrate that the major growth-promoting signaling pathway in MCL, the Rb-E2F cell cycle regulatory pathway, is affected upon SOX11-induced overexpression and mediates the observed growth reduction.

## Discussion

Recent data have suggested a functional role for the developmentally associated transcription factor SOX11 outside the CNS [2,3,6], and this marker has been shown to be expressed in specific subtypes of B cell lymphomas [1,4,18], as well as in solid tumors [6]. Furthermore, the potential regulatory role of SOX11, indicated by its association with clinical features, such as survival [2,3,6], require further investigations to understand the underlying biology and potential clinical application of this marker.

Gene expression can be regulated by epigenetic mechanisms, such as DNA methylation of CpG islands in the 5' promoter region [20,21]. Methylation-mediated silencing of various genes, often tumor suppressor genes, is a well studied phenomenon in many cancers [22], since such methylation provides a growth advantage for the malignant cells. An increasing number of hypermethylated genes have been reported in lymphomas [23-30], where they have been shown to be involved in various cellular functions, such as cell cycle control [23], cytokine signaling [27], DNA repair and apoptosis [28].

Analysis of the *SOX11 *promoter identified the presence of CpG islands, and bisulfite sequencing demonstrated a strong correlation between promoter methylation status and *SOX11 *mRNA and protein levels in both B cell lymphoma cell lines and primary tumors. Thus, as previously reported, data from cell lines can represent the methylation status of primary malignant tissue well [31]. However, as our experiments illustrate, and also reported by others, the magnitude of methylation is more pronounced in cell lines, since they often display either a full methylation or no methylation at all [32]. Altogether, it is clear that the absence of SOX11 expression is tightly coupled to a methylated promoter in primary tumor samples, however no specific determinative CpG position could be identified as most alleles were either fully methylated or unmethylated.

In addition to investigating the cause of differential *SOX11 *expression, we also explored the relationship between SOX11 and cellular growth, as a correlation with improved survival has been reported [1,3]. SOX11 function in the CNS has previously been assessed, using siRNA in a mouse neuroblastoma cell line and in cultured mouse dorsal root ganglia neurons, where *SOX11 *was shown to modulate the levels of several other unrelated mRNAs involved in cell survival and death, suggesting an anti-apoptotic role [33]. In contrast, SOX11 was recently shown to prevent gliomagenesis *in vivo *by induced neuronal differentiation and abolished expression of oncogenic *plagl1 *[9]. Recent clinical studies have shown both a positive and negative correlation of SOX11 to survival and further studies are consequently needed to fully explore the clinical implications of this marker [2,3,6]. In the present study, transient knock-down experiments confirm a growth regulatory role for SOX11 in B cell malignancies, as decreased levels result in increased proliferation in several *in vitro *models of MCL. To further clarify if SOX11 is the limiting factor in a signaling cascade or if SOX11 possibly exhibits a master regulatory property, we overexpressed *SOX11 *in different B cell lymphoma cells lines with variable degree of wild-type *SOX11 *expression. Overexpression was achieved in all cell lines, independent of the original *SOX11 *status, and resulted in an increase in SOX11 protein. Of note, all cell lines were functionally affected and their growth rates were significantly reduced. The direct effect on proliferation upon increasing SOX11 levels confirms that SOX11 is a master growth regulator.

It is known since previously that proliferation in MCL is partly driven by an overexpression of *CCND1*, which leads to an increased ability to pass through the G1/S cell cycle checkpoint [34]. Using global gene expression analysis we now show that the *CCND1*-related Rb-E2F pathway [19,35] is affected by the increased level of SOX11. Among others, up-regulation of the CDKN2A locus, coding for p16^INK4A ^and p14^ARF^, is seen already at 24 h of ectopic SOX11-overexpression. The CDKN2A locus encode both p16^INK4A ^and p14^ARF^, but the proteins have no sequence homology due to alternative reading frames [36]. The precise mechanisms of CDKN2A regulation and induction is unknown, but it has been shown that p16^INK4A ^and p14^ARF ^levels responds to, (i) external stress signals, (ii) Jun, Ets and Id families of transcription factors and (iii) hyperproliferative signals from for example Ras, Myc or deregulated E2F, as reviewed by Lowe and Sherr [37]. Thus, it remains to be determined how SOX11 induces changes to the Rb-E2F signaling pathway although it is of major biological interest that this MCL-associated protein affects the same signaling pathway as *CCND1*.

In agreement with the observed decrease in proliferation both p16^INK4A ^and p14^ARF ^possess anti-proliferative functions [36,38] and constitute two of the three pathways that control the G1/S transition of the cell cycle and are thus targeted in many tumors [39]. E2F1 is down-regulated at 48 h and is one of the down-stream targets of p14^ARF^. It has been suggested that E2F1 is the limiting factor for cell cycle transition [36], and the decrease may thus directly contribute to the observed growth reduction.

Furthermore, TGF-β is up-regulated at 24 h and induces expression of down-stream genes, including TGFβR1, SMAD2/3 and TAB1/2 at 48 h. Of note, we have previously demonstrated that MCL cell lines are responsive to TGF-β-mediated decrease in proliferation when the corresponding receptor is available [40]. Thus, the up-regulation of TGF-βR seen at 48 h emphasizes the need for a functional receptor to achieve TGF-β-mediated growth reduction. Consequently, it is likely that the anti-proliferative activity of TGF-β together with reduced levels of E2F1 is directly involved in growth reduction induced by ectopic SOX11-overexpression. The anti-proliferative effect of TGF-β is widely known and its dual role as both tumor suppressor and pro-metastatic mediator makes it an interesting target for intervention [41-43].

## Conclusions

We have for the first time shown that the expression of the transcription factor SOX11 is inversely correlated to specific promoter methylation in hematopoietic malignancies and that SOX11 has growth regulatory properties. Gene Chip analysis revealed that TGF-β and components of the Rb-E2F proliferative pathway, including E2F1 and the CDKN2A locus, are involved in the SOX11-induced growth reduction. Thus, based on both experimental and previous clinical observations we suggest that SOX11 can act as a master regulator of lymphoid tumor cell growth.

## Methods

### Cultivation of cell lines

Nineteen lymphoma cell lines were used to study *SOX11 *including eight MCL, four follicular lymphoma (FL), three diffuse large B cell lymphoma (DLBCL), three Burkitt lymphoma (BL) and one acute monocytic leukemia (MONO-L), as shown in Table 1. Most cell lines were provided and authenticated by DSMZ (Table 1). All cell lines were cultured in RPMI-1640 medium (HyClone, Sout Logan, UT) supplemented with 10% (v/v) fetal bovine serum (Invitrogen Gibco, Carlsbad, CA, USA) and 2 mM L-Glutamine (Sigma-Aldrich, St. Louis, MO, USA), hereafter referred to as R10 medium, except ULA which was cultured in 45% optiMEM (HyClone), 45% IMDM (HyClone) supplemented with 10% (v/v) fetal bovine serum (Invitrogen).

### Collection and purification of primary samples

Lymphocytes were isolated from four MCLs, five FLs and one DLBCL through density centrifugation, as previously described [44]. All five FL samples and two of the MCL samples (MCL1 and MCL6) were purified by positive selection, using a CD19 specific antibody (clone HD37, DAKO, Glostrup, Denmark) coupled to Dynabeads Pan Mouse IgG magnetic beads (Invitrogen Dynal), according to the protocol of the manufacturer. Flow cytometry was used to determine the purity of MCL 3 and 4 and the DLBCL. Patient material information is shown in Additional File 1: Table S1.

### DNA methylation analysis

MethPrimer http://www.urogene.org/methprimer/index1.html[45] was used to analyze the 2000 bp region directly upstream of the *SOX11 *transcription start site (the *SOX11 *promoter region) for the presence of CpG islands. Using the MethPrimer default algorithm, three CpG islands were identified as >200 bp regions with G and C contents >50% and Observed/Expected CpG-rates of >0.6. One additional CpG island was detected when the region size constraint was lowered to 100 bp without altering the other criteria (Figure 1). The methylation status of the 5'-promoter region was determined by sodium bisulfite sequencing [46] of the 213 bp CpG island located -435 to -222 bp upstream of the *SOX11 *transcription start site. Briefly, total genomic DNA was extracted from five million cells per cell line or primary samples, using QIAamp DNA MINI kit (QIAgen) according to the protocol of the manufacturer. To convert unmethylated cytosine to uracil, we performed bisulfite conversion of 0.5 - 1 μg of DNA with EpiTect Bisulfite Kit (QIAgen). The CpG island was amplified from bisulfite converted DNA, using primers 5'-AGA GAG ATT TTA ATT TTT TGT AGA AGG A-3'and 5'-CCC CCT TCC AAA CTA CAC AC-3' and Platinum Taq DNA polymerase (Invitrogen). PCR products were both directly sequenced as well as ligated into the vector pCR.21-TOPO (Invitrogen) for clonal analysis. Sequencing was performed by Eurofins MWG Operon (Ebersberg, Germany and GATC Biotech (Konstanz, Germany). Quality control of methylation data was performed in a standardized manner, using the BiQ Analyzer software [47], http://biq-analyzer.bioinf.mpi-inf.mpg.de/index.php. Images of CpG methylation for figures 3A-C were constructed using the BDPC web server [48], using output files from BiQ Analyzer. All amplicons included in the study had, (i) bisulfite conversion rates above 95% for unmethylated non-CpG C:s to T:s, and (ii) sequence similarity above 90% compared to the original genomic sequence.

### Nucleofection of cell lines with SOX11-specific siRNA or overexpression plasmid

The Amaxa protocol http://www.lonzabio.com/protocols.html for nucleofection of suspension cell lines was followed, using program 0-017 and Cell Line Nucleofector Solution T (Amaxa Biosystems, Cologne, Germany). For the knock-down experiments, 5 × 10^6 ^cells were mixed with 50 pmol of siRNA (Ambion, Austin, TX, USA) in each reaction and a scrambled sequence and GFP-producing plasmid were used as controls. The sequences of the siRNAs in the pool targeting the *SOX11 *gene can be found in Additional File: Table S3. For the overexpression experiments, 5 × 10^6 ^cells were mixed with 2 μg of OmicsLink™Expression Clone for SOX11 (EX-M0425-M60, the sequence can be found in the Additional File 1: Table S2) in each reaction and a GFP control vector was used as a control (both from GeneCopoeia, Germantown, MD, USA).

### RNA isolation and Real Time-qPCR analysis of wt and SOX11-knocked/overexpressed cell lines

The relative quantity (RQ) of *SOX11 *mRNA in various wt cell lines was identified using Real Time-quantitative PCR (RT-qPCR). The cells were lysed and cDNA synthesis performed using the Fast SYBR Green Cells-to-C_T _kit (Applied Biosystems), according to the protocol of the manufacturer. Briefly, 10^4 ^cells were washed in PBS, lysed and treated with DNase. Lysates were reversely-transcribed and cDNA amplified in three technical replicates with the following primer specific either for *SOX11 *or the house-keeping gene GAPDH (250 nM, MWG, High-Point, NC, USA); GAPDH: 5'-TGGTATCGTGGAAGGACTC-3' and 5'-AGTAGAGGCAGGGATGATG-3', *SOX11-t*: 5'-GGTGGATAAGGATTTGGATTCG-3' and 5'-GCTCCGGCGTGCAGTAGT-3'. q-PCR conditions were as follows: enzyme activation 20 seconds at 95°C, PCR cycle denaturation for 3 seconds at 95°C and anneal/elongation 30 seconds at 60°C run on a Fast 7500 real-time qPCR system (Applied Biosystems). All samples were run in triplicates. In the reverse-transcription, a control sample was run containing lysate but no reverse transcriptase (RT), to check for background amplification of genomic *SOX11 *and *GAPDH*. A ΔC_T _> 4 for *GAPDH *(+RT) and *GAPDH *(-RT) was achieved for all cell lines. Similarly, the ΔC_T _for *SOX11 *(+RT) and *SOX11 *(-RT) was used as a qualitative control to determine if *SOX11 *was expressed or not. Generally, all samples with a ΔC_T __(SOX11+RT, SOX11-RT) _< |2| was considered negative and the RQ was set to 0.01 for those samples. Finally, RQ is calculated as 2^-(ΔΔCT(SOX11-GAPDH)) ^comparing each cell line to GRANTA-519. All the error bars related to qPCR data have been calculated using the standard error (SE) with a 95% confidence level.

For the over-expression experiments the Fast SYBR Green Cells-to-C_T _kit (Applied Biosystems) was used for lysis of 0.5-1.0*10^5 ^cells and subsequent cDNA synthesis as described above. The *SOX11-t *and GAPDH primer sets were used for amplification

In the knock-down experiments RNA isolation was carried out, using Trizol (Invitrogen) as previously described [44]. The cDNA synthesis was performed, as outlined in the RevertAid™First Strand cDNA Synthesis kit-protocol (Fermentas). 1 μg of RNA was mixed with 0.2 μg random hexamer primers, and a reverse transcriptase was added to produce cDNA. Samples for RT-qPCR were prepared following the iQ™SYBR Green Supermix protocol (Bio-Rad, Hercules, CA, USA). The concentration of cDNA was 1.25-2.5 μg/l. The primers were as above but primers for Eg5 were included and a different set of *SOX11 *primers were used as follows: *SOX11-u*: 5'-CCAGGACAGAACCACCTGAT-3' and 5'-CCCCACAAACCACTCAGACT-3', Eg5: 5'-GTTTGGCCATACGCAAAGAT-3' and 5' - GAGGATTGGCTGACAAGAGC-3'. The RT-qPCR was run in triplicate, using a 2-Step Amplification and melt-curve program (Bio-Rad) with GAPDH as the endogenous control.

### Protein purification and quantification

0.5-2 × 10^6 ^cells were harvested, washed and placed in 200 μl lysis-buffer (1% NP40/Protease Inhibitor cocktail (Roche, Basel, Switzerland) in PBS) and incubated on ice for 30 min. Centrifugation (16,000 × g at 4°C for 30 min) was used to remove cell debris. Protein concentrations were determined using the BCA Kit for Protein Determination (Sigma-Aldrich) with BSA as a standard (0.08 - 0.4 mg/ml). The samples were mixed with BCA working reagent, incubated at 37°C for 30 min, and absorbance measured at 562 nm.

### Western Blot analysis of *SOX11*-knockdown and differential expression

Protein lysates, 3 or 7 μg for knock-down experiments, 3.5 μg for overexpression experiments and 32 μg for wild-type expression in nineteen lymphoma cell lines and fifteen primary specimens were run on NuPAGE 10% Bis-Tris gels (Invitrogen) under reducing conditions for ~45 min at 130 V. Separated proteins were blotted onto PVDF membranes, Amersham Hybond-P (GE Healthcare, Uppsala, Sweden) for 30 min (15 V) and blocked over night in 5% milk PBS. SOX11 protein expression was verified using anti-Sox-11^C-term ^(Figure 2, 3, 4 or Sox-11^N-term ^(Figure 5) antibodies, as previously described [1,4]. Primary antibodies Eg5 (Becton Dickinson, Franklin Lakes, NJ, USA) or GAPDH (Abcam) were used as loading control. HRP-labeled swine anti-rabbit antibody or rabbit anti-mouse antibody (DAKO) was used as secondary antibody and detection was made with SuperSignal West Femto Max Sensitivity Substrate (Pierce Biotechnology Inc., Rockford, IL), according to the protocol of the manufacturer. Blots were developed, using the SuperSignal West Femto Maximum Sensitivity Substrate (Nordic Biolabs, T228;by, Sweden) and detected with either with ECL Hyperfilm (GE Healthcare) in Kodak X-OMAT 1000 processor (Kodak Nordic AB, Upplands V228;sby, Sweden) or using a chemiluminescence scanner and CCD camera (Bio-Rad Laboratories, Hercules, California).

### Assessment of proliferation

All proliferation assays were quantified using Methyl-3H-Thymidine (MTT) incorporation, as previously described [49]. 50 000 cells were plated in triplicates for each sample. For all proliferation results, the ± 1 standard deviation (SD) is shown.

### Isolation of tRNA for GeneChip Whole Transcript analysis

GRANTA-519 and JEKO-1 cells were nucleofected with SOX11-GFP or control GFP-containing vector, as described above, and 100 000 cells were washed once in PBS and lysed in 300 μl Trizol (Life Technologies, Gaithersburg, MD). The RNA was precipitated using chloroform/isopropanol extraction and dissolved in 6 μl of RNA-free H_2_O. The integrity and quantity of the RNA was assessed using Agilent 2100 bioanalyzer with the RNA 6000 Nano LabChip^® ^reagent set (Agilent Technologies Inc., Santa Clara, CA, USA) and stored at -20°C. For all arrays 300 ng tRNA starting material was used for the first strand cDNA synthesis which was followed by amplification, fragmentation, labeling, hybridization, washing and scanning, all performed according to the Affymetrix standard protocol "GeneChip^® ^Whole Transcript (WT) Sense Target Labeling Assay User manual, P/N 701880 Rev.5 (Affymetrix Inc., Santa Clara, CA). The labeled cRNA was hybridized to the Human Gene ST 1.0 arrays (Affymetrix Inc.). All arrays passed the initial quality control using assessment of hybridization, amplification controls and noise levels as defined by Affymetrix Inc.

### Bioinformatic analysis of Gene Chip data

Raw data was extracted from Human Affymetrix Gene arrays, HuGene ST 1.0, using the Command Console software package (Affymetrix). Quantile normalization using RMA and quality control was done in the Expression Console 1.0 software (Affymetrix). Normalized data was imported into Gene Spring GX 11.0 (Agilent Technologies Inc.) for fold change analysis. Fold change analyses for 24 and 48 h treatments were performed for GRANTA 519 and JEKO-1, comparing SOX11 overexpression to control vector. For each of the cell lines, genes that had a 1.2 fold change comparing SOX11 to control vector, at either 24 h or 48 h were selected. Genes that were differentially expressed in both cell lines were selected for further analysis using Ingenuity Pathway Analysis (Ingenuity Systems).

## Competing interests

The authors declare that they have no competing interests.

## Authors' contributions

EG performed all epigenetic experiments and wrote parts of the manuscript. SS performed siRNA/overexpression experiments and wrote parts of the manuscript. EA performed the overexpression studies. DB was involved in the design of the epigenetic analysis. MD and MJ provided patients material and clinical data. CB was involved in the design of the study, interpretation of the results and writing of the manuscript. SE was responsible for the design of the study, interpretation of data and writing of the manuscript. All authors approved of the final manuscript.

## Supplementary Material

Additional file 1 **Supplementary Tables and Figure**. Additional file 1 contains the following tables and figure. Table S1. Primary patient samples used for epigenetic analysis. Table S2. CDS sequence for the SOX11 OmicsLink™Expression Clone (EX-M0425-M60). Table S3. DNA sequences for the *SOX11 *-targeting siRNAs. Figure S1. Full image of the canonical pathway "Molecular Mechanisms in Cancer" for 24 h (A) and 48 h (B) of SOX11-overexpression. Click here for file

Additional file 2 **Differentially regulated transcripts (n = 4861) in MCL cell lines upon SOX11-overexpression**. Contains all transcripts deregulated (1.2 fold change) upon SOX11-overexpression in GRANTA-519 and JEKO-1. Click here for file
