# Supplementary material for: SOX11 expression correlates to promoter methylation and regulates tumor growth in hematopoietic malignancies
Source: Mol Cancer. 2010 Jul 12;9:187. doi: 10.1186/1476-4598-9-187 (PMC2913986; doi:10.1186/1476-4598-9-187)
Supplement: Additional file 1 — Supplementary Tables and Figure. Additional file 1 contains the following tables and figure. Table S1. Primary patient samples used for epigenetic analysis. Table S2. CDS sequence for the SOX11 OmicsLink™Expression Clone (EX-M0425-M60). Table S3. DNA sequences for the SOX11 -targeting siRNAs. Figure S1. Full image of the canonical pathway "Molecular Mechanisms in Cancer" for 24 h (A) and 48 h (B) of SOX11-overexpression. [file 1476-4598-9-187-S1.DOC]

**Additional File 1 - Supplementary Tables and Figure**

**Table S1. Primary patient samples used for epigenetic analysis**

| **Sample type** | **Age** | **Sex** | **Purification method*** | **Purity**** |
| --- | --- | --- | --- | --- |
| MCL1 | 57 | M | CD19-coupled Dynabeads |  |
| MCL3 | 62 | M |  | >80% |
| MCL4 | na | K |  | >95% |
| MCL6 | 70 | M | CD19-coupled Dynabeads |  |
| FL1 (grade 2) | 56 | F | CD19-coupled Dynabeads |  |
| FL2 (grade 1) | 69 | F | CD19-coupled Dynabeads |  |
| FL3 (grade 3) | 76 | F | CD19-coupled Dynabeads |  |
| FL4 (grade 3) | 85 | M | CD19-coupled Dynabeads |  |
| FL5 (grade 3) | 62 | F | CD19-coupled Dynabeads |  |
| DLBCL | 44 | M |  |  |

* All samples purified using Ficoll-Isopaque centrifugation

**measured as CD19 positive, viable cells in flow cytometry

na – information not available

**Table S2.** CDS sequence for the SOX11 OmicsLink^TM^ Expression Clone (EX-M0425-M60)

atggtgcagcaggcggagagcttggaagcggagagcaacctgccccgggaggcgctggac

acggaggagggcgaattcatggcttgcagcccggtggccctggacgagagcgacccagac

tggtgcaagacggcgtcgggccacatcaagcggccgatgaacgcgttcatggtatggtcc

aagatcgaacgcaggaagatcatggagcagtctccggacatgcacaacgccgagatctcc

aagaggctgggcaagcgctggaaaatgctgaaggacagcgagaagatcccgttcatccgg

gaggcggagcggctgcggctcaagcacatggccgactaccccgactacaagtaccggccc

cggaaaaagcccaaaatggacccctcggccaagcccagcgccagccagagcccagagaag

agcgcggccggcggcggcggcgggagcgcgggcggaggcgcgggcggtgccaagacctcc

aagggctccagcaagaaatgcggcaagctcaaggcccccgcggccgcgggcgccaaggcg

ggcgcgggcaaggcggcccagtccggggactacgggggcgcgggcgacgactacgtgctg

ggcagcctgcgcgtgagcggctcgggcggcggcggcgcgggcaagacggtcaagtgcgtg

tttctggatgaggacgacgacgacgacgacgacgacgacgagctgcagctgcagatcaaa

caggagccggacgaggaggacgaggaaccaccgcaccagcagctcctgcagccgccgggg

cagcagccgtcgcagctgctgagacgctacaacgtcgccaaagtgcccgccagccctacg

ctgagcagctcggcggagtcccccgagggagcgagcctctacgacgaggtgcgggccggc

gcgacctcgggcgccgggggcggcagccgcctctactacagcttcaagaacatcaccaag

**Table S3. DNA sequences for the *SOX11*^a^ -targeting siRNAs.**

| **Sequence** | **Sense 5´→3´** | **Antisense 5´→3´** |
| --- | --- | --- |
| **SOX11.1 (pool)** | CAAGUAUGUUGGUACGUUAuu | UAACGUACCAACAUACUUGuu |
|  | GAUAAGAUGUCGUGACGCAuu | UGCGUCACGACAUCUUAUCuu |
|  | CCUCUAGGCUCCUCGAAGAuu | UCUUCGAGGAGCCUAGAGGuu |
|  | GUUUGAAGCUUGUCGGUCUuu | AGACCGACAAGCUUCAAACuu |

^a)^ nucleotides written in small letters are overhangs

**Figure S1.** (see legend below)

**A**


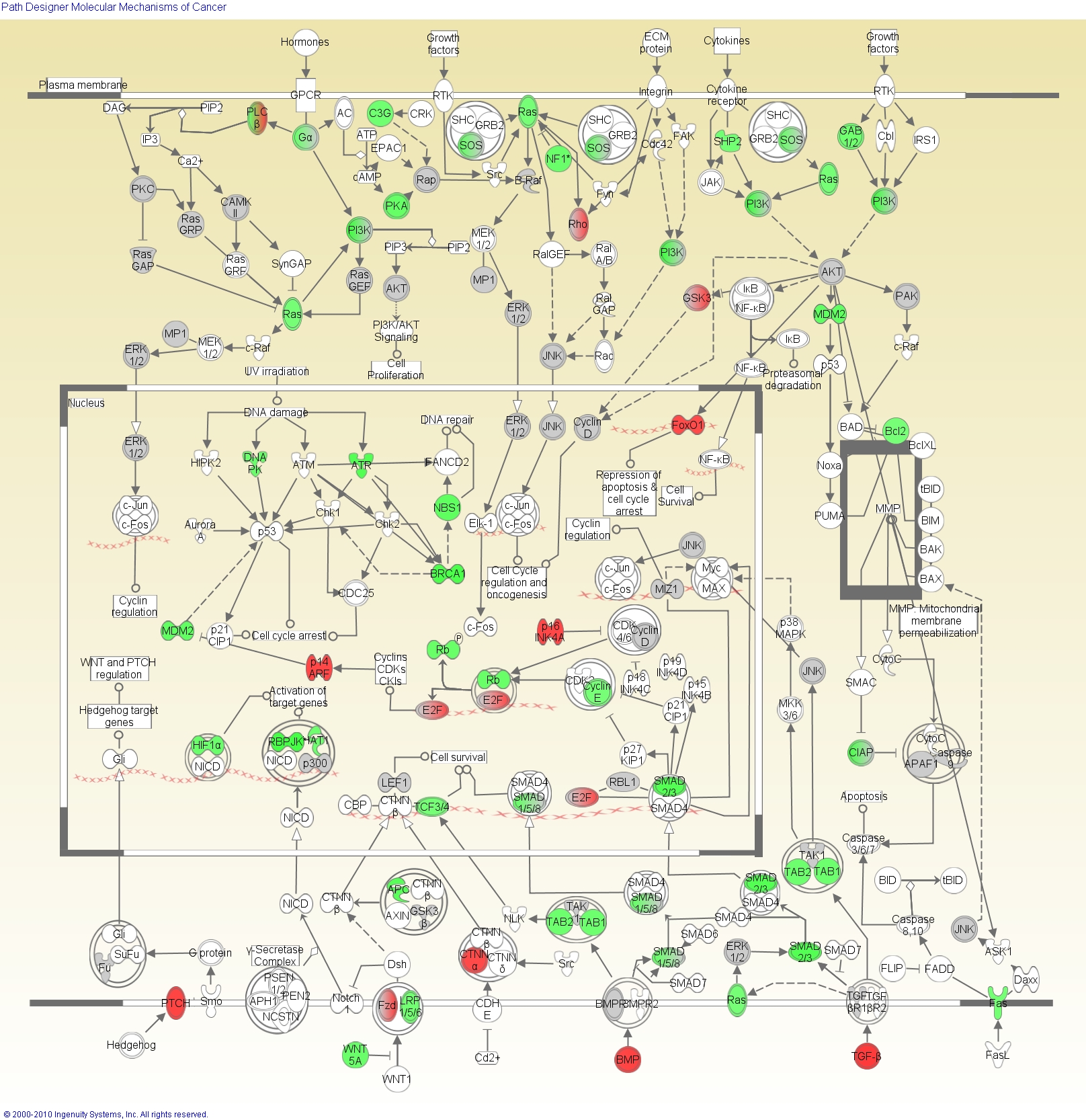


**B**


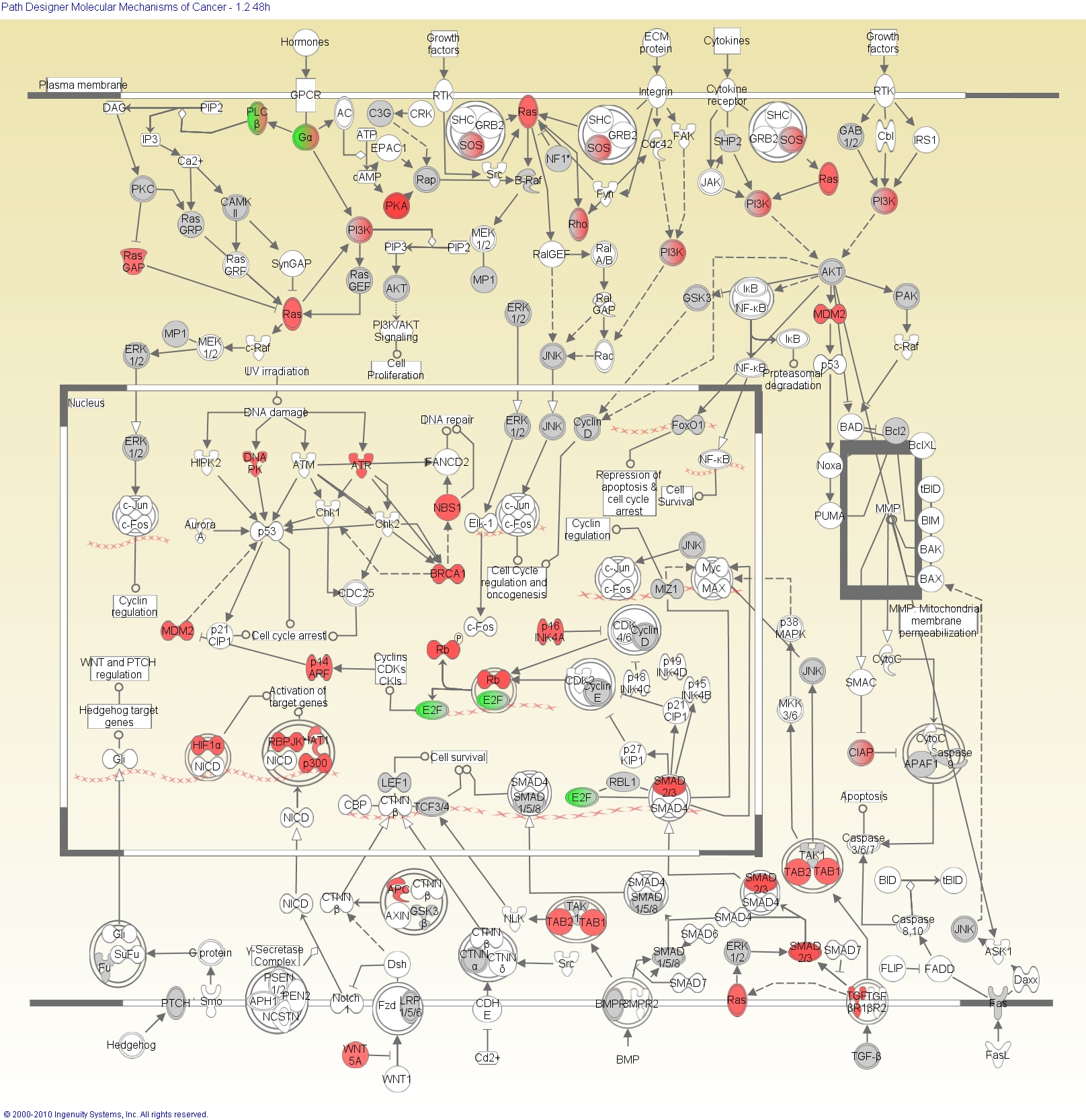


**Figure S1. Full image of the canonical pathway “Molecular Mechanisms in Cancer” for 24h (A) and 48h (B) of SOX11-overexpression.** IPA analysis identified the canonical pathway “Molecular Mechanisms in Cancer” as associated with the 3647 identified genes among the 4861 differentially regulated Gene Chip transcripts comparing ectopic SOX11 overexpression and control vector in MCL cell lines. The differentially regulated genes are marked in red or green when the mean fold change for GRANTA-519 and JEKO-1 was ≥1.2 or ≤1.2, respectively as shown for 24h (A) and 48h (B) of SOX11-overexpression. The remaining differentially regulated genes, including genes with different kinetics or regulation in the two cell lines, are marked in grey.
